# Supplementary material for: CRISPR/Cas9 modified An. gambiae carrying kdr mutation L1014F functionally validate its contribution in insecticide resistance and combined effect with metabolic enzymes
Source: PLoS Genet. 2021 Jul 6;17(7):e1009556. doi: 10.1371/journal.pgen.1009556 (PMC8284791; doi:10.1371/journal.pgen.1009556)
Supplement: S1 Text — Fig A: Strategy for the construction of the CRISPR/Cas9 donor plasmid. The nucleotide sequence of the central part (600bp) of the An. gambiae vgsc (Gene ID: AGAP004707) fragment (1,600bp in total) used to construct the CRISPR/Cas9 donor plasmid for HDR is depicted. Light orange areas correspond to exons 18 and 19. Position 2,422,652 (based on the AgamP3 reference sequence, chromosome arm 2L) where an A>T transversion generates the TTA―›TTT codon alteration creating mutation L1014F is marked with an asterisk. Underlined sequences correspond to the selected CRRISPR/Cas9 targets (gRNAs 1,2,3 and 4). Green areas mark the PAM (-NGG) triplets for each gRNA in the target sequence. Red letters show the SNPs introduced in the donor sequence. These include silent mutations in the gRNA target site to avoid cleavage of the donor plasmid from Cas9 and the A>T transversion creating mutation L1014F. Letters below red letters show the original sequence. Sequencing of the region in individuals from the Kisumu-F/F strain revealed that silent mutations introduced in the target sequences of gRNA’s 2,3 and 4 were retained while those of gRNA 1 were not. Fig B: Comparison of knock-down and mortality. The percentage of mosquitoes being knocked-down (immobile or unable to stand or take off) immediately after exposure to standard WHO assays (1h exposure time) was recorded, as well as the mortality 24h later based on standard WHO criteria (2). Error bars represent the SD (At least 3 replicates of 20, 2–5 day old female mosquitoes each were used per strain). Table A: Summary of screening G1 progeny to identify positive transformants. Table B: Primers used. (DOCX) [file pgen.1009556.s001.docx]

**S1 Text - Supporting Information**

**CRISPR/Cas9 strategy for generating Kisumu-F/F**

Originally the CRSPR/Cas9 strategy we followed included the use of two gRNAs (combination of gRNA 1 and 3 or 1 and 4 in Fig A) that would cut at two sites flanking the TTA codon for amino acid L1014. This strategy had previously been used successfully to introduce target-site resistance mutations in *Drosophila* [1] . However, after multiple rounds of injections we didn’t retrieve mutants and changed the approach using only one gRNA (number 2).

**
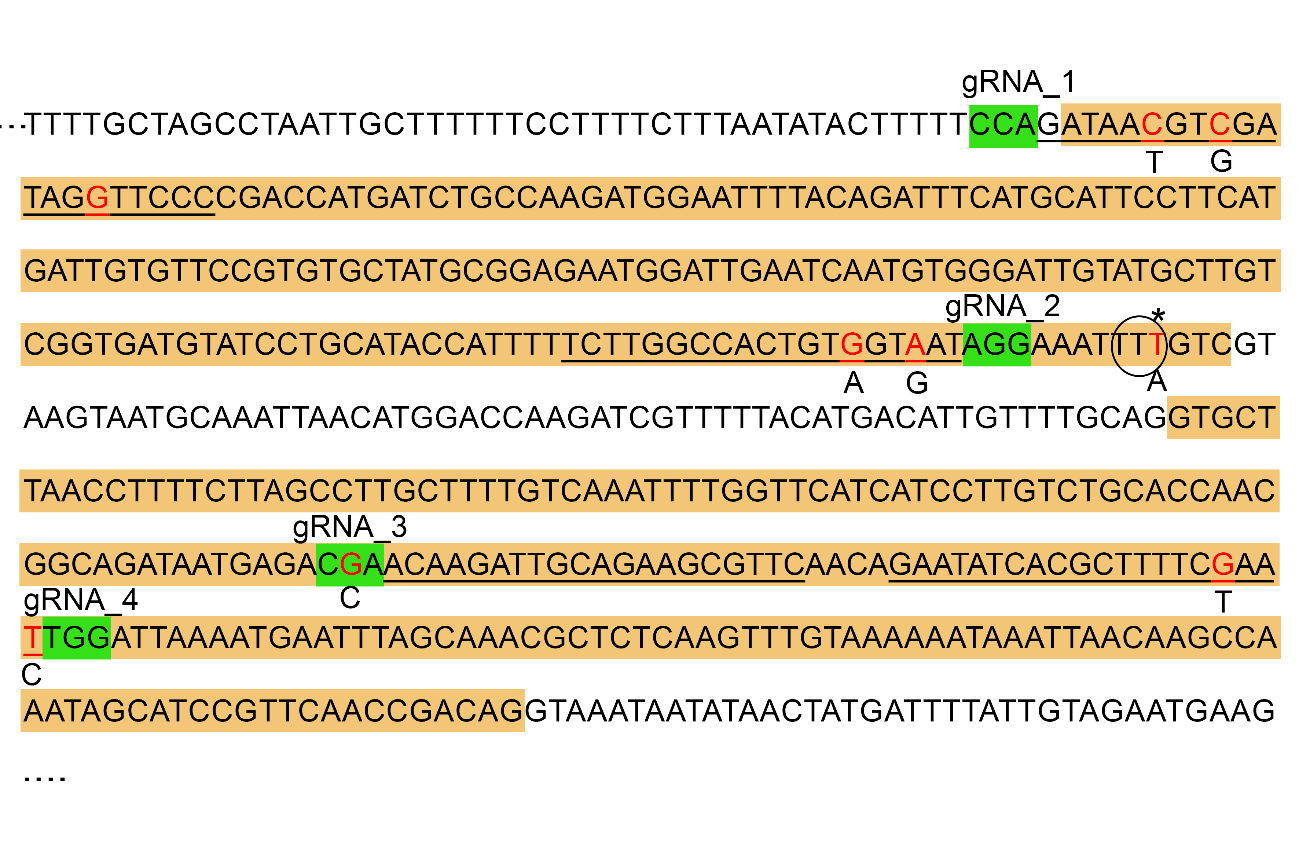
**

**Fig A**: Strategy for the construction of the CRISPR/Cas9 donor plasmid. The nucleotide sequence of the central part (600bp) of the *An. gambiae* *vgsc* (Gene ID : AGAP004707) fragment (1,600bp in total) used to construct the CRISPR/Cas9 donor plasmid for HDR is depicted. Light orange areas correspond to exons 18 and 19. Position 2,422,652 (based on the AgamP3 reference sequence, chromosome arm 2L) where an A>T transversion generates the TTA―›TTT codon alteration creating mutation L1014F is marked with an asterisk. Underlined sequences correspond to the selected CRRISPR/Cas9 targets (gRNAs 1,2,3 and 4). Green areas mark the PAM (-NGG) triplets for each gRNA in the target sequence. Red letters show the SNPs introduced in the donor sequence. These include silent mutations in the gRNA target site to avoid cleavage of the donor plasmid from Cas9 and the A>T transversion creating mutation L1014F. Letters below red letters show the original sequence. Sequencing of the region in individuals from the Kisumu-F/F strain revealed that silent mutations introduced in the target sequences of gRNA’s 2,3 and 4 were retained while those of gRNA 1 were not.


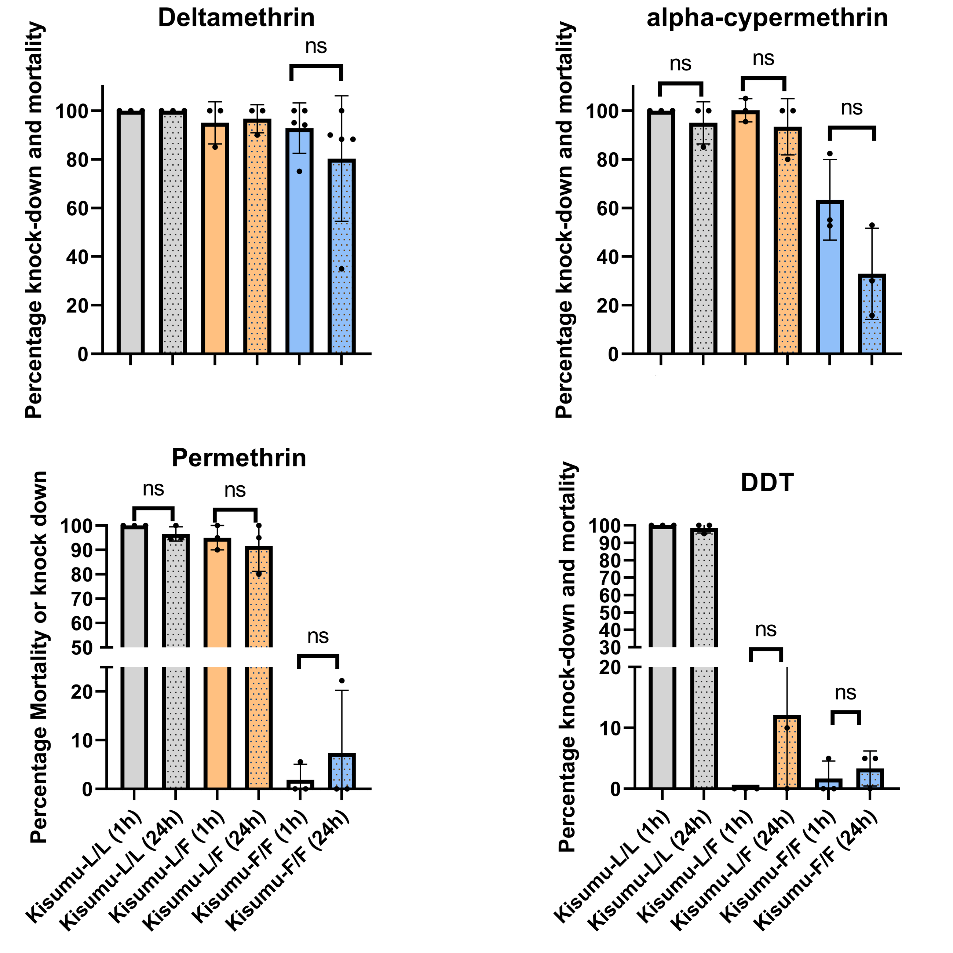


**Fig B**: Comparison of knock-down and mortality. The percentage of mosquitoes being knocked-down (immobile or unable to stand or take off) immediately after exposure to standard WHO assays (1h exposure time) was recorded, as well as the mortality 24h later based on standard WHO criteria [2]. Error bars represent the SD (At least 3 replicates of 20, 2-5 day old female mosquitoes each were used per strain).

**Table A**: Summary of screening G_1_ progeny to identify positive transformants.

| Individual | Number of G_1_ progeny screened | Number of transformed G_1_ progeny |
| --- | --- | --- |
| G_0_ female 1 | 30 | 0 |
| G_0_ female 2 | 28 | 0 |
| G_0_ female 3 | 26 | 0 |
| G_0_ female 4 | 16 | 0 |
| G_0_ female 5 | 22 | 4 (18%) |
| Kisumu female 1 | 10 | 0 |
| Kisumu female 2 | 10 | 0 |
| Kisumu female 3 | 10 | 0 |
| Kisumu female 4 | 4 | 0 |
| Kisumu female 5 | 10 | 0 |
| Kisumu female 6 | 10 | 0 |
| Kisumu female 7 | 4 | 0 |
| Kisumu female 8 | 4 | 0 |
| Kisumu female 9 | 10 | 0 |
| Kisumu female 10 | 10 | 0 |
| Kisumu female 11 | 10 | 0 |
| Kisumu female 12 | 10 | 0 |
| Kisumu female 13 | 6 | 0 |
| Kisumu female 14 | 10 | 0 |
| Kisumu female 15 | 10 | 0 |
| Kisumu female 16 | 10 | 0 |
| Kisumu female 17 | 10 | 0 |
| Kisumu female 18 | 10 | 0 |
| Kisumu female 19 | 10 | 0 |

G0 females were injected as embryos with a CRISPR/Donor plasmid mix and showed transient expression of RFP. Kisumu Females were crossed with G_0_ males that were injected as embryos with a CRISPR/Donor plasmid mix and showed transient expression of RFP.

**Table B**: Primers used

| Primer | Sequence (5’-3’) |
| --- | --- |
| L1014F_gRNA1_F | TGCTGGGAATCTATCCACATTATC |
| L1014F_gRNA1_R | AAACGATAATGTGGATAGATTCCC |
| L1014F_gRNA2_F | TGCTGTCTTGGCCACTGTAGTGAT |
| L1014F_gRNA2_R | AAACATCACTACAGTGGCCAAGAC |
| L1014F_gRNA3_F | TGCTGAACGCTTCTGCAATCTTGT |
| L1014F_gRNA3_R | AAACACAAGATTGCAGAAGCGTTC |
| L1014F_gRNA4_F | TGCTGAATATCACGCTTTTCTAAC |
| L1014F_gRNA4_R | AAACGTTAGAAAAGCGTGATATTC |
| L1014ext_seqF1 | GCTGTTCGGAAAGAACTATGTCG |
| L1014ext_seqR1 | GATATACATGGACATACGCCTTTGC |
| L1014Fint_seqF2 | GATCTGCCAAGATGGAATTTTACAGA (used for sequencing) |
| L1014Fint_seqR3 | CAAGGCTAAGAAAAGGTTAAGCAC (used for sequencing) |

1. Douris V, Steinbach D, Panteleri R, Livadaras I, Pickett JA, Van Leeuwen T, et al. Resistance mutation conserved between insects and mites unravels the benzoylurea insecticide mode of action on chitin biosynthesis. Proc Natl Acad Sci U S A. 2016;113(51):14692-7.

2. World Health Organization, Test Procedures for Insecticide Resistance Monitoring in Malaria Vector Mosquitoes. (World Health Organization, Geneva, Switzerland, ed. 2, 2016).
