# Supplementary material for: CRISPR/Cas9 modified An. gambiae carrying kdr mutation L1014F functionally validate its contribution in insecticide resistance and combined effect with metabolic enzymes
Source: PLoS Genet. 2021 Jul 6;17(7):e1009556. doi: 10.1371/journal.pgen.1009556 (PMC8284791; doi:10.1371/journal.pgen.1009556)
Supplement: Attachment — Submitted filename: Responses to reviewers.docx [file pgen.1009556.s004.docx]

We would like to thank the editor and all reviewers for their positive response to our submitted paper. We have provided answers to their comments below. We have also changed ‘’interaction’’ to ‘’combined effect’’ in the title to be consistent with the term used in the main text.

Kind regards,

Linda Grigoraki and Hilary Ranson

**Comments to the Authors and Responses provided**

Reviewer #1: Acceptable for publication. Would have been nice to see LD50 data for the insecticides.

**Response:** Measurement of insecticide resistance intensity can be done either by determining the Lethal Time 50 (LT50, as done here) or the Lethal Dose 50 (LD50), as mentioned by the reviewer. We chose to calculate the LT50 values for several reasons. A) We wanted to use the WHO toxicity bioassay, as it is the most widely followed and standardized assay to determine resistance in *Anopheles gambiae* mosquitoes. As this assay involves the use of insecticide impregnated papers of a standard dose, we varied the exposure time and determined the LT50 values. B) It has also proven difficult to obtain reliable LC50 data for susceptible strains, like the Kisumu, as shown in the literature (e.g with the CDC bottle assay, Bagi et al., 2015 Malaria J). C) The use of the standard WHO toxicity assay and the provided LT50 values will make it easier to compare our results to other published data on *An. gambiae* mortality levels after insecticide exposure, given the widespread use of these insecticide treated papers.

Reviewer #2: This manuscript describes the use of genome editing to introduce the pyrethroid resistance mutation L1014F into the VGSC of Anopheles gambiae. The impact of this on insecticide sensitivity and organismal fitness was assessed. Furthermore the interaction of this mutation with a metabolic mechanism, over-expression of glutathione transferase Gste2, was investigated.

The manuscript is very well written and the study innovative and important. The kdr mutation has been linked to resistance in An gambiae for more than two decades, however, the precise level of resistance conferred by this mechanism, in isolation or combination with other mechanisms, has remained unclear. This study emphatically demonstrates its importance in conferring resistance while also uncovering fitness costs associated with the mutations presence.

The results are thoroughly and carefully discussed and any potential limitations/confounding effects (i.e. from off-target effects of CRISPR-CAS) noted.

In summary I recommend this paper is accepted and the following typos corrected in production.

Line 192: change: Mutation L1014F carries fitness costs. To ‘The L1014F mutation carries fitness costs’.

**Response**: done

Line 261: correct the grammer in the following sentence: The resistance observed for Kisumu-F/F against permethrin and deltamethrin are very similar to the levels of insensitivity mutation L1014F provides to Ae. aegypti VGSCs expressed in the in vitro Xenopus oocyte system (8-fold for permethrin and 14-fold for deltamethrin) (29).

**Response**: Done.

Reviewer #3: In this paper, Grigoraki et al. generate an Anopheles gambiae transgenic line bearing a kdr mutation to further validate that kdr contributes to insecticide resistance. Using CRISPR/cas9 system, they edit the genome of a susceptible mosquito strain to introduce single nucleotide polymorphism (kdr mutation L1014F). They further test the mortality of this line using different insecticide compounds and confirm kdr mutation L1014F increases resistance to insecticides. Overexpression of gste2, a detoxification enzyme, in the kdr mutant line increase the resistance phenotype. Finally, they analyse the effect of the mutation on mosquito fitness. This paper is well written, clear and conclusions supported by the data. However, I do not think that this study reveals novel methods nor biological insights as it only confirms previous studies showing kdr is involved in resistance to insecticides and therefore, may be better suited for a more specific journal.

**Response**: The results presented in our study are not just another confirmation that the L1014F mutation is involved in insecticide resistance, but rather reveal its relative contribution to resistance which remained elusive and is of fundamental importance for insecticide resistance monitoring and management. The generation of a genome modified An. gambiae line that carries the L1014F mutation in an otherwise fully susceptible background gives us the opportunity to study the effect of this mutation on a variety of phenotypes, including fitness costs and insecticide resistance (presented here), with confidence that the observations made are not affected by differences in the genetic background of the populations/strains compared. In addition, this is the first time CRISPR is used to introduce a single mutation in mosquitoes, which requires a different approach in screening for positive transformants and establishing the genome modified line compared to previous works using fluorescent markers. Thus, our study provides a development of the existing CRISPR methodology in non-model organisms and gives answers to critical questions about the role of L1014F in insecticide resistance. Furthermore the availability of the Kisumu-F/F line will enable future studies both on the impact of this mutation on other traits (several colleagues from other groups have expressed their interest in using the line to look into complex traits that can be substantially impacted by genetic background differences) and its combined impact with other mutations.

Minor comments
Why is there a lot of variation in F/F mosquitoes mortality after ITN exposure (Fig 3) with some mosquito groups showing a mortality above 90%, ie are susceptible?

**Response**: although there is some variation in mortality of F/F mosquitoes after ITN exposure in some replicates, the difference between the lines is clear and statistically significant. Some variation is commonly observed in replicates of toxicity bioassays.

The resistance to deltamethrin varies a lot as well (eg fig2 with 70-80% mortality vs fig4 with less than 20%). How can this be?

**Response**: The exposure times are different in the experiments presented in Figure 2 (1hour exposure) and figure 4 (25min exposure). This is the reason for the different percentages in mortality reported.

**List of changes made to the manuscript:**

-We changes in the title the word ”interaction” to “combined effect”

-We changed line 194 to : “The L1014F mutation carries fitness costs.”

-we changed lines 263-265 to: “Permethrin and deltamethrin resistance observed in Kisumu-F/F is very similar to the levels of insensitivity L1014F confers to *Ae. aegypti* VGSCs expressed in the Xenopus oocyte system (8-fold for permethrin and 14-fold for deltamethrin)”

-We changed throughout the manuscript the word figure to fig to be consistent with the format requested by Plos Genetics

-We added all the raw data from the fitness cost experiments in the Supporting Information dataset 1

-We provided all legends for supplementary figures and files at the end of the manuscript as requested
